# Supplementary material for: Antibiotic Use, Incidence and Risk Factors for Orthopedic Surgical Site Infections in a Teaching Hospital in Madhya Pradesh, India
Source: Antibiotics (Basel). 2022 May 31;11(6):748. doi: 10.3390/antibiotics11060748 (PMC9220190; doi:10.3390/antibiotics11060748)
Supplement: Supplementary file 1 [file antibiotics-11-00748-s001.zip › antibiotics-1751305-supplementary.pdf]

SUPPLEMENTARY MATERIALS

Table S1. Orthopedic SSI incidence rates per year in the teaching hospital, Ujjain, central India.

Year	Incidence Rate (%)
2013 (Aug-Dec)	15.5
2014	6.25
2015	6.45
2016 (Jan-Apr)	3.65

Table S2. Patient-related potential risk factors for orthopedic surgical site infections distributed by sex in the teaching hospital, Ujjain, central India.

	**All operated patients**			**SSI Patients**			**Non-SSI Patients**			**P-value**
	**Total n=1205**	**Male n=838**	**Female n=367**	**Total SSI n=91**	**Male n=80**	**Female n=11**	**Total Non-SSI n=1114**	**Male n=758**	**Female n=356**	**<0.001**
**Age, median (25-75th), years**										
	35 (19-50)	33 (18-50)	40 (22-60)	35 (22-50)	35 (22-50)	40 (16-60)	35 (18-50)	32 (17-50)	40 (23-60)	0.9260
**Age, years, n (%)**										
**≤18**	301 (100)	219 (73)	82 (27)	18 (6)	15 (5)	3 (1)	283 (94)	204 (68)	79 (26)	0.416
**19-60**	760 (100)	533 (70)	227 (30)	64 (8)	58 (8)	6 (1)	696 (92)	475 (63)	221 (29)	**<0.001**
**>60**	144 (100)	86 (60)	58 (40)	9 (6)	7 (5)	2 (1)	135 (94)	79 (65)	56 (39)	0.218
**ASA score, n (%)**										
**ASA I**	1013 (100)	680 (67)	333 (33)	63 (6)	53 (5)	10 (1)	950 (94)	627 (62)	323 (32)	**0.003**
**ASA II**	148 (100)	132 (89)	16 (11)	22 (15)	21 (14)	1 (1)	126 (85)	111 (75)	15 (10)	0.468
**ASA III**	43 (100)	26 (60)	17 (40)	6 (14)	6 (14)	0	37 (86)	20 (46)	17 (40)	0.066
**ASA IV**	1 (100)	0	1 (100)	0	0	0	1 (100)	0	1 (100)	-
**Antibiotic prescribed 14 days before hospital admission, n (%)**										
	73 (100)	60 (82)	13 (18)	17 (23)	16 (22)	1 (1)	56 (77)	44 (60)	12 (17)	0.132
**Previous hospitalisation, n (%)**										
	173 (100)	141 (82)	32 (18)	33 (19)	31 (18)	2 (1)	140 (81)	110 (64)	30 (17)	**0.046**

Pearson's Chi-squared and Fisher's exact test (for values with expected frequency less than five) were used to compare the categorical variables and the Mann-Whitney test was used to compare the medians of continuous variables. P-values for categorical variables were calculated by comparing the distribution of sex of SSI and non-SSI patients. P-values for continuous variables were calculated by comparison of SSI and non-SSI patients. Significant p-values (<0.05) are marked in bold. Percentages are calculated by division with number of all operated patients. SSI=surgical site infection, ASA=American Society for Anesthesiologists.

Table S3. Surgery-related potential risk factors for orthopedic surgical site infections distributed by sex in the teaching hospital, Ujjain, central India.

	All operated patients			SSI Patients			Non-SSI Patients			p-value
	Total n=1205	Male n=838	Female n=367	Total SSI n=91	Male n=80	Female n=11	Total Non-SSI n=1114	Male n=758	Female n=356	
**Type of wound[a], n (%)**										
Closed	1034 (100)	702 (68)	332 (32)	64 (6)	54 (5)	10 (1)	970 (94)	648 (63)	322 (31)	**0.004**
Compound fracture	37 (100)	31 (84)	6 (16)	9 (24)	9 (24)	0	28 (76)	22 (60)	6 (16)	0.302
Clean	3 (100)	3 (100)	0	0	0	0	3 (100)	3 (100)	0	-
Contaminated	23 (100)	17 (74)	6 (26)	3 (13)	3 (13)	0	20 (87)	14 (61)	6 (26)	0.539
**Nature of surgery[a], n (%)**										
Elective	1113 (100)	770 (69)	343 (31)	80 (7)	70 (6)	10 (1)	1033 (93)	700 (63)	333 (30)	**<0.001**
Emergency	17 (100)	11 (65)	6 (35)	2 (12)	1 (6)	1 (6)	15 (88)	10 (59)	5 (29)	1.000
**Duration of surgery[a], min**										
≤60	425 (100)	301 (71)	124 (29)	40 (9)	34 (8)	6 (1)	385 (91)	267 (63)	118 (28)	**0.038**
61-120	375 (100)	255 (68)	120 (32)	22 (6)	20 (5)	2 (1)	353 (94)	235 (63)	118 (31)	**0.017**
>120	208 (100)	146 (70)	62 (30)	13 (6)	10 (5)	3 (1)	195 (94)	136 (66)	59 (28)	0.759
**Hair removal method[a], n (%)**										
Shaving	1057 (100)	731 (69)	326 (31)	76 (7)	66 (6)	10 (1)	981 (93)	665 (63)	316 (30)	**0.001**
Clipping	2 (100)	2 (100)	0	0	0	0	2 (100)	2 (100)	0	-
**Preoperative shower, n (%)**										
	267 (100)	181 (68)	86 (32)	42 (16)	35 (13)	7 (3)	225 (84)	146 (55)	79 (29)	**0.019**
**Preoperative LOS, median (25-75th), days**										
	5 (3-9)	5 (3-8)	6 (3-9)	4 (2-8)	4 (1-8)	5 (3-11)	5 (3-9)	5 (3-8)	6 (3-9)	0.0843
**Preoperative LOS[a], days**										
1-3	325 (100)	231 (71)	94 (29)	24 (7)	19 (6)	5 (1)	301 (93)	212 (65)	89 (28)	0.364
4-7	379 (100)	265 (70)	114 (30)	28 (7)	26 (7)	2 (0)	351 (93)	239 (63)	112 (30)	**0.005**
8-15	312 (100)	202 (65)	110 (35)	16 (5)	14 (4)	2 (1)	296 (95)	188 (60)	108 (35)	0.060
>15	90 (100)	63 (70)	27 (30)	9 (10)	7 (8)	2 (2)	81 (90)	56 (62)	25 (28)	0.719
**Postoperative LOS, median (25-75th), days**										
	8 (3-14)	8 (3-14)	9 (4-14)	13 (4-21)	13 (3-21)	13 (7-27)	8 (3-14)	8 (3-14)	9 (4-14)	**<0.001**
**Postoperative LOS[a], days**										
1-3	223 (100)	156 (70)	67 (30)	7 (3)	7 (3)	0	216 (97)	149 (67)	67 (30)	0.106
4-7	239 (100)	165 (69)	74 (31)	8 (3)	5 (2)	3 (1)	231 (97)	160 (67)	71 (30)	0.706
8-15	440 (100)	287 (65)	153 (35)	28 (6)	24 (5)	4 (1)	412 (94)	263 (60)	149 (34)	**0.023**
>15	203 (100)	152 (75)	51 (25)	33 (16)	29 (14)	4 (2)	170 (84)	123 (61)	47 (23)	0.078
**Oxygen support, n (%)**	1031 (100)	716 (69)	315 (31)	73 (7)	62 (6)	11 (1)	958 (93)	654 (63)	304 (30)	**0.003**
**Blood transfusion, n (%)**	405 (100)	274 (68)	131 (32)	27 (7)	26 (7)	1 (0)	378 (93)	248 (61)	130 (32)	**<0.001**
**Drain, n (%)**	41 (100)	30 (73)	10 (24)	8 (20)	7 (18)	1 (2)	33 (80)	24 (58)	9 (22)	0.653
**Implant, n (%)**	297 (100)	221 (74)	76 (26)	49 (17)	44 (15)	5 (2)	248 (84)	177 (60)	71 (24)	**0.007**
**Antibiotic prescription, n (%)**										
	1133 (100)	791 (70)	342 (30)	91 (8)	80 (7)	11 (1)	1042 (92)	711 (68)	331 (32)	**<0.001**
**PAP, n (%)**	840 (100)	580 (69)	260 (31)	42 (5)	37 (4)	5 (1)	798 (95)	543 (65)	255 (30)	**0.006**
**Antibiotic during hospital stay before PAP, n (%)**										
	258 (100)	192 (74)	66 (26)	43 (17)	36 (14)	7 (3)	215 (83)	156 (60)	59 (23)	0.126
**Duration of antibiotic treatment before PAP, days**										
1-7	186 (100)	138 (74)	48 (26)	29 (16)	25 (14)	4 (2)	157 (84)	113 (61)	44 (23)	0.079
8-14	44 (100)	30 (68)	14 (32)	8 (18)	7 (16)	1 (2)	36 (82)	23 (52)	13 (30)	0.194
>14	28 (100)	24 (86)	4 (14)	6 (21)	4 (14)	2 (7)	22 (79)	20 (72)	2 (7)	0.191
**Postoperative antibiotic, n (%)**										
	1036 (100)	720 (69)	316 (31)	75 (7)	64 (6)	11 (1)	961 (93)	656 (63)	305 (29)	**0.002**
**Duration of postoperative antibiotic, days**										
1-7	440 (100)	307 (70)	133 (30)	17 (4)	14 (3)	3 (1)	423 (96)	293 (67)	130 (29)	0.191
8-14	374 (100)	250 (67)	124 (33)	24 (6)	20 (5)	4 (1)	350 (94)	230 (62)	120 (32)	0.056
>14	248 (100)	179 (72)	69 (28)	36 (15)	32 (13)	4 (2)	212 (85)	147 (59)	65 (26)	**0.010**
**Antibiotic duration, median (25-75th), days**										

	12 (4-16)	12 (4-16)	11 (4-15)	24 (8-36)	23 (7-34)	31 (14-40)	11 (4-15)	10 (4-15)	11 (4-16)	**<0.001**
**Total antibiotic duration[a], days**										
1-7	384 (100)	276 (72)	108 (28)	21 (5)	21 (5)	0	363 (95)	255 (67)	108 (28)	**0.002**
8-14	319 (100)	208 (65)	111 (35)	19 (6)	14 (4)	5 (2)	300 (94)	194 (61)	106 (33)	0.424
> 14	391 (100)	285 (73)	106 (27)	50 (13)	44 (11)	6 (2)	341 (87)	241 (62)	100 (25)	**0.010**

[a] For the variables where the number of patients does not correspond to the total number of patients in the group, that information for the rest of the patients is missing in the data record. Pearson's Chi-squared and Fisher's exact test (for values with expected frequency less than five) were used to compare the categorical variables and the Mann-Whitney test was used to compare the medians of continuous variables. P-values for categorical variables were calculated by comparing the distribution of sex of SSI and non-SSI patients. P-values for continuous variables were calculated by comparison of SSI and non-SSI patients. Significant p-values (<0.05) are marked in bold. Percentages are calculated by division with number of all operated patients. SSI=surgical site infection, PAP=perioperative antibiotic prophylaxis, LOS= length of stay.
